# Design, Preclinical Evaluation, and Clinical Translation of ^68^Ga-FAPI-LM3, a Heterobivalent Molecule for PET Imaging of Nasopharyngeal Carcinoma

**DOI:** 10.2967/jnumed.123.266183

**Published:** 2024-03

**Authors:** Liang Zhao, Yizhen Pang, Jianyang Fang, Jianhao Chen, Yangfan Zhou, Long Sun, Hua Wu, Zhide Guo, Qin Lin, Haojun Chen

**Affiliations:** 1 Department of Nuclear Medicine and Minnan PET Center, Xiamen Key Laboratory of Radiopharmaceuticals, First Affiliated Hospital of Xiamen University, School of Medicine, Xiamen University, Xiamen, China;; 2 School of Clinical Medicine, Fujian Medical University, Fuzhou, China;; 3 Xiamen Key Laboratory of Radiation Oncology, Department of Radiation Oncology, Xiamen Cancer Center, First Affiliated Hospital of Xiamen University, School of Medicine, Xiamen University, Xiamen, China;; 4 State Key Laboratory of Molecular Vaccinology and Molecular Diagnostics and Center for Molecular Imaging and Translational Medicine, School of Public Health, Xiamen University, Xiamen, China; and; 5 Xiamen Key Laboratory of Rare Earth Photoelectric Functional Materials, Xiamen Institute of Rare Earth Materials, Haixi Institute, Chinese Academy of Sciences, Xiamen, China

**Keywords:** fibroblast activation protein, somatostatin receptor 2, heterobivalent molecule, nasopharyngeal carcinoma, PET

## Abstract

Extensive research has been conducted on radiolabeled fibroblast activation protein (FAP) inhibitors (FAPIs) and p-Cl-Phe-cyclo(d-Cys-Tyr-d-4-amino-Phe(carbamoyl)-Lys-Thr-Cys)d-Tyr-NH_2_ (LM3) peptides for imaging of FAP and somatostatin receptor 2 (SSTR2)–positive tumors. In this study, we designed and synthesized a FAPI-LM3 heterobivalent molecule radiolabeled with ^68^Ga and evaluated its effectiveness in both tumor xenografts and patients with nasopharyngeal carcinoma (NPC). **Methods:** The synthesis of FAPI-LM3 was based on the structures of FAPI-46 and LM3. After radiolabeling with ^68^Ga, its dual-receptor–binding affinity was evaluated in vitro and in vivo. Preclinical studies, including small-animal PET and biodistribution evaluation, were conducted on HT-1080-FAP and HT-1080-SSTR2 tumor xenografts. The feasibility of ^68^Ga-FAPI-LM3 PET/CT in a clinical setting was evaluated in patients with NPC, and the results were compared with those of ^18^F-FDG. **Results:** ^68^Ga-FAPI-LM3 showed high affinity for both FAP and SSTR2. The tumor uptake of ^68^Ga-FAPI-LM3 was significantly higher than that of ^68^Ga-FAPI-46 and ^68^Ga-DOTA-LM3 in HT-1080-FAP–plus–HT-1080-SSTR2 tumor xenografts. In a clinical study involving 6 NPC patients, ^68^Ga-FAPI-LM3 PET/CT showed significantly higher uptake than did ^18^F-FDG in primary and metastatic lesions, leading to enhanced lesion detectability and tumor delineation. **Conclusion:** ^68^Ga-FAPI-LM3 exhibited FAPI and SSTR2 dual-receptor–targeting properties both in vitro and in vivo, resulting in improved tumor uptake and retention compared with that observed with monomeric ^68^Ga-FAPI and ^68^Ga-DOTA-LM3. This study highlights the clinical feasibility of ^68^Ga-FAPI-LM3 PET/CT for NPC imaging.

## Visual Abstract

**Figure:**
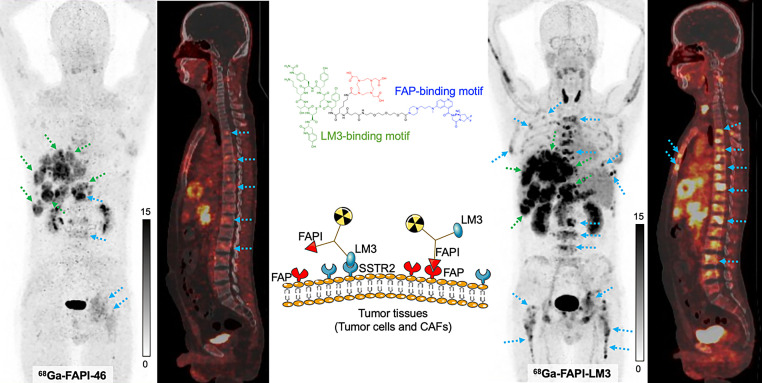

The tumor microenvironment encompasses multiple types of nontumor cells, including cancer-associated fibroblasts, immune cells, and endothelial cells. The tumor microenvironment has attracted significant attention in research on tumor occurrence and development (1). Regarding nasopharyngeal carcinoma (NPC), the Epstein–Barr virus can promote fibrosis and NPC progression by activating signaling of YAP1/fibroblast activation protein (FAP) α in fibroblasts (2). Cancer-associated fibroblasts enhance the survival of irradiated NPC cells through the NF-κB pathway, leading to increased radioresistance (3). Therefore, diagnostic and therapeutic approaches focusing on the tumor microenvironment could be crucial frontiers in NPC. The use of the FAP-targeting inhibitor (FAPI), small ligand (oncoFAP), and cyclic peptide (FAP-2286) has achieved impressive results in tumor diagnosis. However, further improvement is needed to enhance their efficacy in radioligand therapy (4–6).

Within the domain of nuclear medicine, several targets, such as somatostatin receptor 2 (SSTR2), prostate-specific membrane antigen, and FAP, have emerged as prominent subjects of research (7,8). Epstein–Barr virus latent membrane protein 1 can upregulate SSTR2 expression via the NF-κB pathway (9). Both FAP and SSTR2 are important markers in the biology of NPC, as evidenced by the plethora of such PET radiopharmaceuticals, including ^68^Ga-FAPI for imaging cancer-associated fibroblasts and ^68^Ga-DOTATATE for imaging SSTR2 (10,11). For example, over 80% of patients showed positive expression of SSTR2 in both primary and metastatic NPCs (11). In addition to NPC, SSTR2 is expressed in other malignancies, including neuroendocrine tumor, thyroid carcinoma, breast cancer, and meningioma, expanding its potential applications in tumor theranostics (12–14). Apart from agonists, antagonists such as p-Cl-Phe-cyclo(d-Cys-Tyr-d-4-amino-Phe(carbamoyl)-Lys-Thr-Cys)d-Tyr-NH_2_ (LM3) and JR11 are undergoing extensive research for their SSTR2-targeting properties. Unlike SSTR2 agonists, which exhibit a high internalization rate, the antagonist LM3 possesses a high binding affinity to SSTR2 but a low internalization rate (15). Notably, LM3, characterized by low liver uptake and superior lesion-to-background contrast, shows promising potential for peptide-receptor radionuclide therapy (15).

Therefore, developing a heterobivalent molecule targeting both SSTR2 and FAP holds significant importance. In this study, we designed a heterobivalent molecule called FAPI-LM3 and evaluated its preliminary application in preclinical models and patients with NPC. We hypothesized that this heterobivalent peptide can effectively combine the merits of SSTR2 and FAP, resulting in favorable pharmacokinetic characteristics and initial clinical effects.

## MATERIALS AND METHODS

### Chemistry and Radiochemistry

Information regarding the reagents, chemicals, high-performance liquid chromatography, liquid chromatography–mass spectrometry, and flow diagram of the synthesis of FAPI-LM3 are provided in the supplemental materials (available at http://jnm.snmjournals.org (16)). The radiolabeling of FAPI-46, DOTA-LM3, and FAPI-LM3 precursors with ^68^Ga was performed following similar procedures. In brief, 25 nmol of the precursor in 1 mL of sodium acetate buffer (0.25 M, pH 8.2–8.3) was allowed to react with 4 mL of ^68^Ga solution (1.3 GBq in 0.6 M HCl) at 100°C for 15 min. For clinical imaging, the final product was passed through a 0.22-μm Millipore filter for sterilization in each ^68^Ga-FAPI-LM3 preparation process. The stability of ^68^Ga-FAPI-LM3 was determined by incubating the product in phosphate-buffered saline and fetal bovine serum at 37°C and analyzing it via radio–high-performance liquid chromatography after 1 and 2 h of incubation.

### In Vitro Characterization of FAPI-LM3

HT-1080-FAP and HT-1080-SSTR2 cell lines were derived from HT-1080 cells (obtained from the China National Infrastructure of Cell Line Resource) and stably transfected with human FAP and SSTR2, respectively, following our previously established protocol (17). Information on cell resources, transfection, and culture is provided in the supplemental materials. For in vitro studies, the cells were seeded in 24-well plates and cultured in a routine medium until they reached approximately 80% confluence. During the experiment, the medium was replaced with a fetal bovine serum–free medium. In the cellular uptake test, different cells (HT-1080-FAP, HT-1080-SSTR2, HT-1080-FAP–plus–HT-1080-SSTR2, C666-1, or U87 cells) were treated with ^68^Ga-FAPI-LM3 with or without 10 nmol of unlabeled precursor (FAPI-46, DOTA-LM3, or FAPI-46 plus DOTA-LM3) and incubated for 60 min. For FAP radioligand binding assays, HT-1080-FAP cells were incubated with unlabeled FAPI-LM3 or FAPI-46 (8.16 × 10^−5^ to 10^−13^ M, *n* = 3) using ^68^Ga-FAPI-46 as the radioligand. Similarly, for the SSTR2 receptor binding assay, HT-1080-SSTR2 cells were incubated with unlabeled FAPI-LM3 or DOTA-LM3 (8.16 × 10^−5^ to 10^−13^ M, *n* = 3) using ^68^Ga-DOTA-LM3. After a 60-min incubation period, the free tracer was removed using phosphate-buffered saline before measurement. The cells were lysed with 0.5 mL of 1 M NaOH, and radioactivity was subsequently gauged using a γ-counter (Wizard 2480; PerkinElmer Inc.).

### Small-Animal PET Imaging and Biodistribution Studies

The Animal Care and Use Committee of Xiamen University approved all animal studies. Six-week-old BALB/c nude mice were obtained from Beijing Vital River Laboratory Animal Technology Co. In total, 5 × 10^6^ tumor cells (2.5 × 10^6^ HT-1080-FAP and 2.5 × 10^6^ HT-1080-SSTR2) in 100 μL of phosphate-buffered saline were subcutaneously injected into the right shoulder of each mouse. Tumor-bearing mice (3/group) were intravenously injected with approximately 7.4 MBq of ^68^Ga-FAPI-LM3, ^68^Ga-FAPI-46, or ^68^Ga-DOTA-LM3. Subsequent static PET scans were obtained at intervals of 0.5, 1, 2, and 4 h after injection using an Inveon small-animal PET scanner (Siemens). In the blocking experiment, approximately 60 nmol of the unlabeled precursor (FAPI-46, DOTA-LM3, or FAPI-46 plus DOTA-LM3) were simultaneously injected with ^68^Ga-FAPI-LM3. The PET images were reconstructed iteratively using 3-dimensional OpMAP 256 (PET reconstruction protocol [.pPetRcn]; Siemens Healthineers AG) and converted to percentage injected dose per gram of tissue (%ID/g) by delineating the regions of interest. For the biodistribution studies, approximately 1.48 MBq of ^68^Ga-FAPI-LM3, ^68^Ga-FAPI-46, ^68^Ga-DOTA-LM3, or ^68^Ga-FAPI-LM3 with unlabeled precursor were injected into tumor-bearing mice (3/group), and different groups of mice were euthanized at the scheduled time points after injection.

### PET/CT Imaging in Healthy Volunteers and Patients with NPC

The clinical study was approved by the institutional review board of the First Affiliated Hospital of Xiamen University and registered at ClinicalTrials.gov (NCT05873777). Written informed consent was obtained from all healthy volunteers and patients. Selection of the voluntary cohort was based on a set of inclusion and exclusion criteria. The inclusion criteria were adults (>18 y) with no known history of chronic disease or cancer. The exclusion criteria were pregnancy or breastfeeding. Each participant received a 3.0–3.7 MBq/kg dose of ^68^Ga-FAPI-LM3 on the basis of our prior study related to another heterobivalent agent (18). Adverse events were monitored for 4 h after the injection of ^68^Ga-FAPI-LM3. The PET/CT scans and reconstruction protocols are presented in the supplemental materials. Doses were calculated using OLINDA/EXM software (version 1.1) (19). Six patients underwent paired ^68^Ga-FAPI-LM3 and ^18^F-FDG PET/CT imaging for comparison, whereas 1 patient underwent paired ^68^Ga-FAPI-LM3 and ^68^Ga-FAPI-46 PET/CT imaging for comparison. For quantitative analysis, SUV_max_ and SUV_mean_ were used to measure uptake by normal organs and tumor tissues. Delayed ^68^Ga-FAPI-LM3 PET/CT scans were obtained at 3 h after injection in 6 patients to analyze the in vivo distribution pattern. For our PET/CT study, the inclusion of a healthy cohort was pivotal in understanding the tracer’s normal biodistribution, which helps in distinguishing pathologic from physiologic uptake. Furthermore, we attempted to test both tracers in a single patient to generate head-to-head comparison data, aiding in the direct comparison of their diagnostic efficacies.

### FAP and SSTR2 Immunohistochemistry in NPC

Tissue microarrays of human NPC (HNasN110su01) were purchased from Shanghai Outdo Biotech Co. The Ethics Committee of the Shanghai Outdo Biotech Co. approved the study. Continuous sections of tumor tissue microarrays were used to ensure consistency in verifying different biomarkers in immunohistochemistry experiments. For immunohistochemistry analysis of paraffin-embedded areas, the BenchMark ULTRA (Ventana Medical Systems) automated slide stainer was used to stain cells with the anti-FAP antibody (ab218164; Abcam) or anti-SSTR2 antibody (ZA-0587; ZAGB-BIO) according to the manufacturer’s recommendation. The sections were visualized, and images were captured using a Leica microscope. FAP and SSTR2 expression was semiquantitatively evaluated using the H-score method (20). Negative expression was defined as an H-score of less than 10.

### Statistical Analysis

All statistical analyses were conducted using SPSS 22.0 software (IBM). Mean values were compared using the Student *t-*test, whereas SUVs derived from ^18^F-FDG and ^68^Ga-FAPI-LM3 PET/CT were compared using the Wilcoxon matched-pairs signed-rank test. Differences were considered statistically significant at a *P* value of less than 0.05 in a 2-tailed test.

## RESULTS

### Synthesis and Radiolabeling

**FIGURE 1.**
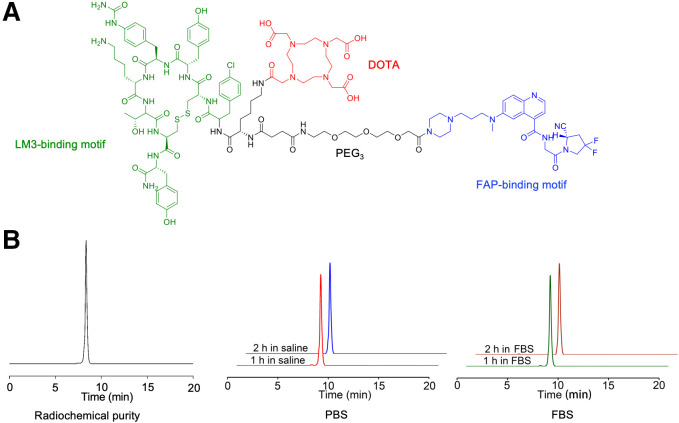
(A) Chemical structure of FAPI-LM3. (B) Radiochemical purity and stability of ^68^Ga-FAPI-LM3 via radio–high-performance liquid chromatography analysis. FBS = fetal bovine serum; PBS = phosphate-buffered saline; PEG_3_ = polyethylene glycol 3.

Polyethylene glycol 3 groups incorporating a heterobivalent of FAPI-46 and LM3 and the chelator DOTA were synthesized (Fig. 1A; Supplemental Fig. 1). Radiolabeling of ^68^Ga-FAPI-LM3 was achieved at an activity concentration of approximately 74 MBq/mL (molar activity, 29 GBq/μmol), with over 95% radiochemical purity after purification (Fig. 1B). High-performance liquid chromatography analysis showed that ^68^Ga-FAPI-LM3 exhibited high stability for up to 2 h, with no significant demetallation observed in the presence of phosphate-buffered saline and fetal bovine serum (>99%) (Fig. 1B).

### Selective Binging of Heterobivalent Peptide FAPI-LM3 to Human FAP and SSTR2

**FIGURE 2.**
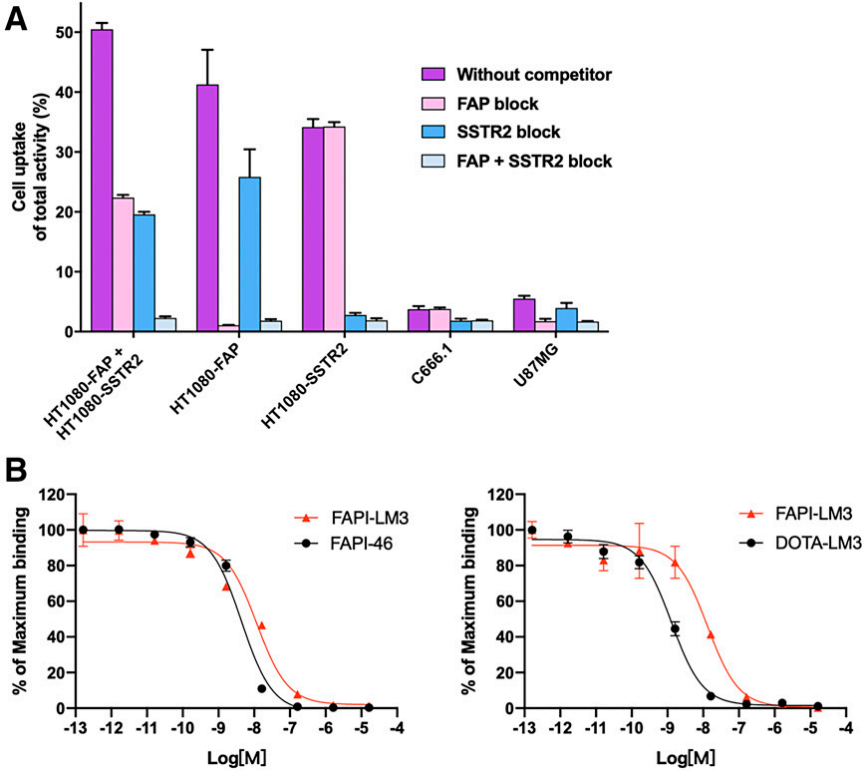
(A) Cell uptake assay of ^68^Ga-FAPI-LM3 and blocking experiments on HT1080-FAP, 1080-SSTR2, HT1080-FAP–plus–1080-SSTR2, C666-1, and U87MG cells. (B) Inhibition of ^68^Ga-FAPI-46 binding to FAP on HT1080-FAP cells by unlabeled FAPI-LM3 and unlabeled FAPI-46 (8.16 × 10^−5^ to 10^−13^ M, *n* = 3, left); inhibition of ^68^Ga-DOTA-LM3 binding to SSTR2 on HT-1080-SSTR2 cells by unlabeled FAPI-LM3 and unlabeled DOTA-LM3 (8.16 × 10^−5^ to 10^−13^ M, *n* = 3, right).

In the cell uptake study, the heterobivalent peptide ^68^Ga-FAPI-LM3 exhibited effective binding to all cell types (Fig. 2). In double-target–positive cells (HT-1080-FAP plus HT-1080-SSTR2), FAP-positive cells (HT-1080-FAP and U87MG), and SSTR2-positive cells (HT-1080-SSTR2 and C666-1), the binding of ^68^Ga-FAPI-LM3 to FAP or SSTR2 was significantly blocked by the corresponding unlabeled agent (all *P* < 0.05, shown in Supplemental Table 1), indicating the specificity of targeting both human FAP and SSTR2. Additional cellular uptake studies were conducted using a standard sample (precursor labeled with natural gallium). Blocking experiments with either natural gallium–labeled or unlabeled precursors showed similar trends (Supplemental Fig. 2).

The binding affinities of FAPI-46 and FAPI-LM3 for FAP were evaluated in HT-1080-FAP cells, whereas the SSTR2-binding affinities of DOTA-LM3 and FAPI-LM3 were evaluated in HT-1080-SSTR2 cells. Comparative analysis of half-maximal inhibitory concentrations indicates that FAPI-LM3 has lower FAP (11.72 vs. 4.39 nM) and SSTR2 (13.21 vs. 1.30 nM; Fig. 2B) binding affinities than does its corresponding counterpart.

### Better Tumor Uptake and Retention with Heterobivalent Molecule FAPI-LM3 Than with Corresponding Monomer in Mice

**FIGURE 3.**
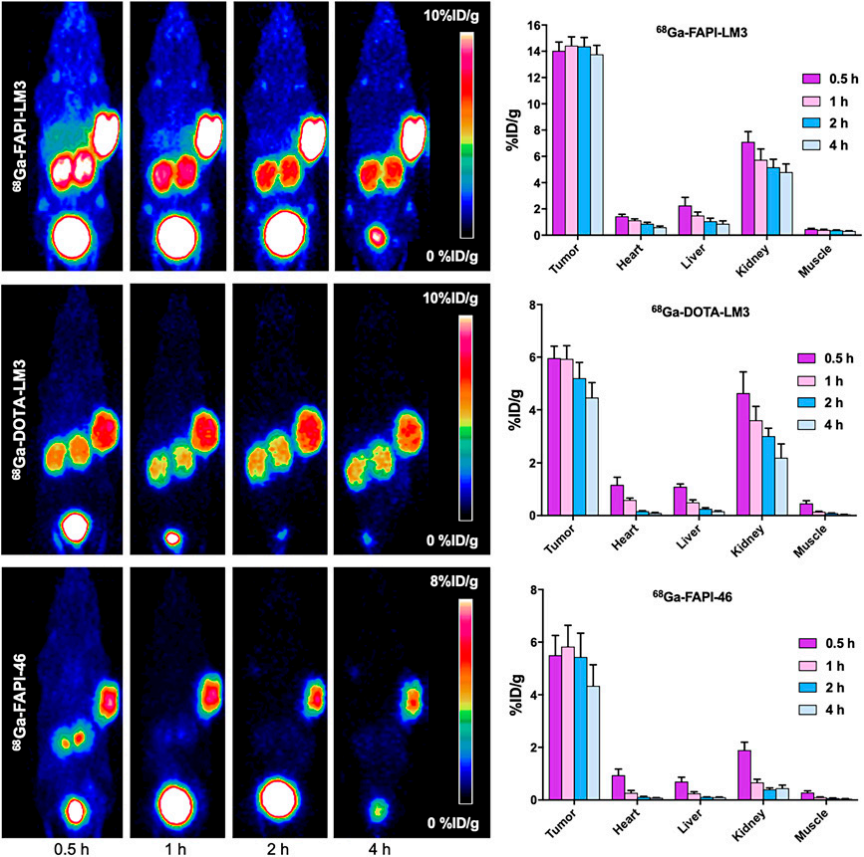
Representative static PET imaging and quantification results in HT1080-FAP–plus–HT1080-SSTR2 tumor-bearing mice with ^68^Ga-FAPI-LM3, ^68^Ga-FAPI-46, and ^68^Ga-DOTA-LM3.

Small-animal PET imaging was performed on a HT-1080-FAP–plus–HT-1080-SSTR2 tumor xenograft model, which is dual-receptor–positive. ^68^Ga-FAPI-LM3 rapidly accumulated in FAP and SSTR2 dual-positive tumors at 0.5 h after injection (14.03 ± 0.47 %ID/g) and remained steady until 4 h after injection (2 h, 14.37 ± 0.68 %ID/g; 4 h, 13.77 ± 0.68 %ID/g) (Fig. 3). The tumor accumulation of ^68^Ga-FAPI-46 remained stable for up to 2 h after injection (5.43 ± 0.91 %ID/g) and then decreased at 4 h after injection (4.33 ± 0.80 %ID/g), whereas tumor uptake of ^68^Ga-DOTA-LM3 decreased from 1 to 4 h (5.93 ± 0.50 vs. 4.46 ± 0.57 %ID/g). Quantitative PET data revealed that tumor uptake of ^68^Ga-FAPI-LM3 was significantly higher than that of ^68^Ga-FAPI-46 and ^68^Ga-DOTA-LM3 at all examined time points. Uptake of ^68^Ga-FAPI-LM3 in the main organs was relatively low and decreased over time. Therefore, ^68^Ga-FAPI-LM3 PET imaging yielded favorable tumor-to-background ratios over time.

**FIGURE 4.**
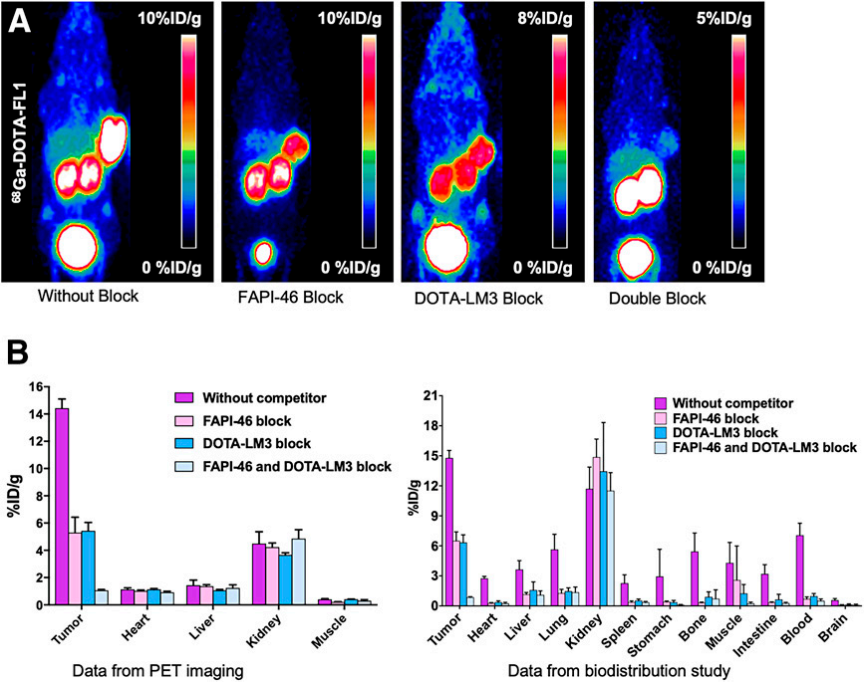
(A) Representative PET imaging of ^68^Ga-FAPI-LM3 and blocking with FAPI-46, DOTA-LM3, or FAPI-46 plus DOTA-LM3 in HT1080-FAP–plus–1080-SSTR2 tumor model. (B) Quantification results of PET imaging and biodistribution studies of ^68^Ga-FAPI-LM3 at 1 h with or without simultaneous injection of unlabeled inhibitors after administration.

The receptor specificity of ^68^Ga-FAPI-LM3 was evaluated through several blocking studies, wherein ^68^Ga-FAPI-LM3 was administered simultaneously with unlabeled FAPI-46, DOTA-LM3, or FAPI-46 plus DOTA-LM3 (Fig. 4). At 1 h after injection, tumor uptake (14.43 ± 0.67 %ID/g) was mostly suppressed when FAPI-46 and DOTA-LM3 were coadministered with ^68^Ga-FAPI-LM3 (1.07% ± 0.06%, 93% blockade). Tumor uptake could be partially blocked by unlabeled FAPI-46 (5.30 ± 1.27 %ID/g, 63% blockade) and DOTA-LM3 (5.43 ± 0.61 %ID/g, 62% blockade).

The ex vivo biodistribution of ^68^Ga-FAPI-LM3 was evaluated in HT-1080-FAP–plus–HT-1080-SSTR2 xenografts at different time points, with results similar to those of the PET studies. ^68^Ga-FAPI-LM3 accumulated mainly in the tumor tissue and remained stable from 1 to 4 h after injection (14.78 ± 0.76 %ID/g at 1 h; 17.68 ± 2.46 %ID/g at 4 h). Biodistribution studies of ^68^Ga-FAPI-46 and ^68^Ga-DOTA-LM3 were also performed for comparison (Supplemental Fig. 3). At 1 h after injection, tumor uptake of ^68^Ga-FAPI-LM3 was significantly higher than that of ^68^Ga-FAPI-46 (14.78 ± 0.76 vs. 8.48 ± 1.75 %ID/g; *P* < 0.001) and ^68^Ga-DOTA-LM3 (14.78 ± 0.76 vs. 7.88 ± 1.10 %ID/g; *P* < 0.001). In addition, high ^68^Ga-FAPI-LM3 uptake was observed in blood. The blocking data in the biodistribution experiment exhibited the same tendency as those of the PET studies (Fig. 4).

### Safety and Radiation Dosimetry of ^68^Ga-FAPI-LM3 in Healthy Volunteers

No adverse events were observed with ^68^Ga-FAPI-LM3 in any healthy volunteers or patients during the injection or at the 4-h follow-up. Supplemental Figure 4 displays representative PET maximum-intensity projection images of a healthy volunteer and biodistribution data on 3 volunteers’ normal organs.

According to the results from OLINDA/EXM, the effective dose of ^68^Ga-FAPI-LM3 was determined to be 1.49 × 10^−2^ mSv/MBq (Table 1), which was comparable to that of ^68^Ga-FAPI-46 (1.80 × 10^−2^ mSv/MBq) (21) but lower than that of ^68^Ga-DOTA-LM3 (2.50 × 10^−2^ mSv/MBq) (15).

**TABLE 1.** ^68^Ga-FAPI-LM3 Dosimetry Summary of Effective Doses Using OLINDA/EXM

Target organ	Mean (mSv/MBq)	SD (mSv/MBq)
Adrenal glands	5.98E–05	2.35E–05
Brain	1.63E–06	4.45E–07
Breasts	3.47E–05	4.15E–06
Gallbladder wall	—	—
LLI wall	4.34E–04	1.01E–04
Small intestine	2.71E–05	1.21E–05
Stomach wall	4.04E–04	4.13E–05
ULI wall	1.53E–05	7.73E–06
Heart wall	—	—
Kidneys	1.08E–04	3.45E–05
Liver	1.28E–03	3.52E–04
Lungs	7.68E–04	1.91E–04
Muscle	1.57E–05	9.87E–06
Ovaries	3.66E–04	1.87E–05
Pancreas	1.23E–04	5.03E–05
Red marrow	7.62E–04	8.49E–05
Osteogenic cells	4.22E–05	4.00E–06
Skin	6.29E–06	8.34E–07
Spleen	1.23E–04	3.57E–05
Thymus	—	—
Thyroid	3.68E–06	1.85E–06
Urinary bladder wall	2.14E–03	9.87E–05
Uterus	2.19E–04	7.60E–05
Effective dose equivalent	1.20E–03	1.03E–03
Effective dose	1.49E–02	2.55E–03

LLI = lower large intestine; ULI = upper large intestine.

### ^68^Ga-FAPI-LM3 PET/CT Imaging in Patients with NPC

In this study, 6 patients were enrolled for initial staging (4 patients) or relapsed detection (2 patients), and their detailed clinical information is provided in Supplemental Table 2. ^68^Ga-FAPI-LM3 and ^18^F-FDG PET/CT imaging was conducted with a median interval of 3 d (range, 1–7 d). ^68^Ga-FAPI-LM3 exhibited durable retention in all lesions up to 3 h after injection. When tumor uptake was compared between the 2 time points, SUV_max_ at the delayed time point (3 h) was significantly higher than that at the routine time point (1 h) for regional lymph node (11.4 vs. 10.1; *P* = 0.031), liver (16.5 vs. 14.3; *P* = 0.046), bone (11.2 vs. 10.4; *P* = 0.003), and distant lymph node (16.9 vs. 14.5; *P* = 0.028) metastases. However, there was no statistical difference in SUV_max_ between 3 and 1 h for primary tumors (Supplemental Table 3).

**FIGURE 5.**
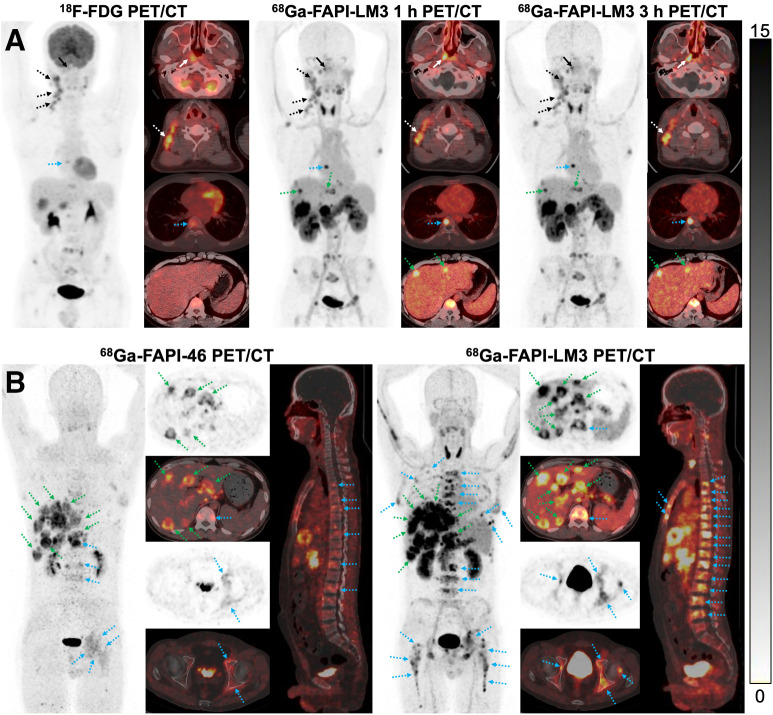
(A) PET/CT imaging findings in 32-y-old treatment-naïve patient with NPC. Both ^18^F-FDG and ^68^Ga-FAPI-LM3 PET/CT showed abnormal activity in primary tumor (solid white arrows), regional lymph node (dotted white arrows), and bone (dotted blue arrows). Additional liver metastases with intense activity (dotted green arrows) were observed on ^68^Ga-FAPI-LM3 PET/CT. However, these lesions were not visualized on ^18^F-FDG PET/CT. (B) PET/CT imaging findings in 44-y-old patient with metastatic NPC. ^68^Ga-FAPI-LM3 PET/CT revealed more lesions than did ^68^Ga-FAPI-46 for lymph node, liver (dotted green arrows), and bone (dotted blue arrows) metastases, with higher uptake.

In the paired ^68^Ga-FAPI-LM3 and ^18^F-FDG PET/CT scans, 5 primary tumors, 34 metastatic lymph nodes, and 37 bone or visceral metastatic lesions were evaluated. The SUV_max_ derived from ^68^Ga-FAPI-LM3 PET/CT was significantly higher than that derived from ^18^F-FDG in the primary tumors (13.8 vs. 9.3, *P* = 0.043), regional lymph node metastases (11.4 vs. 6.6, *P* < 0.001), and distant metastases (14.6 vs. 5.6, *P* < 0.001) (Table 2). Specifically, the SUV_max_ obtained from ^68^Ga-FAPI-LM3 PET/CT was approximately 2–4 times greater than that from ^18^F-FDG PET/CT in liver (16.5 vs. 3.7, *P* = 0.028) and bone (11.2 vs. 4.6, *P* = 0.001) metastases. As a result, ^18^F-FDG missed several metastatic lesions, including regional lymph node (*n* = 3), liver (*n* = 4), bone (*n* = 1), peritoneal (*n* = 2), and retroperitoneal lymph node (*n* = 1) metastases. Interestingly, these ^18^F-FDG–negative lesions could be visualized by ^68^Ga-FAPI-LM3 PET/CT. Representative images from ^18^F-FDG and ^68^Ga-FAPI-LM3 PET/CT are shown in Figure 5A and Supplemental Figure 5. Additionally, 1 patient underwent paired ^68^Ga-FAPI-LM3 and ^68^Ga-FAPI-46 PET/CT for comparison. ^68^Ga-FAPI-LM3 PET/CT showed more lesions than ^68^Ga-FAPI-46 for lymph node, liver, and bone metastases, with higher uptake (Fig. 5B; Supplemental Fig. 6).

**TABLE 2.** Comparison of SUV_max_ on ^68^Ga-FAPI-LM3 and ^18^F-FDG PET/CT Images in Primary and Metastatic Tumors

Tumor	*n*	Median size (cm)	^68^Ga-FAPI-LM3 PET/CT		^18^F-FDG PET/CT		*P* for median SUV_max_*
			Positive tumors (*n*)	Median SUV_max_	Positive tumors (*n*)	Median SUV_max_	
Primary	5	NA	5	13.8 (10.0–21.1)	5	9.3 (6.6–18.4)	0.043
Regional LN mets	34	1.4 (0.8–3.1)	34	11.4 (4.5–19.0)	31	6.6 (2.1–16.0)	<0.001
Liver mets	6	1.7 (1.3–4.0)	6	16.5 (8.6–26.3)	2	3.7 (3.1–10.8)	0.028
Bone mets	15	1.2 (0.8–2.2)	15	11.2 (7.4–18.0)	14	4.6 (2.0–8.2)	0.001
Pleural mets	5	2.3 (1.1–4.0)	5	17.3 (14.6–21.1)	5	7.2 (3.7–12.0)	0.043
Peritoneal mets	4	0.9 (0.7–3.2)	4	13.1 (7.0–18.7)	2	3.4 (1.5–8.6)	0.068
Distant LN mets	7	2.3 (0.9–4.1)	7	16.9 (10.8–22.5)	6	8.2 (2.0–9.0)	0.018
Total^†^	37	1.4 (0.7–4.1)	37	14.6 (7.0–26.3)	29	5.6 (1.5–12.0)	<0.001

* ^68^Ga-FAPI-LM3 vs. ^18^F-FDG.

† Included liver (*n* = 6), bone (*n* = 15), pleura (*n* = 5), peritoneum (*n* = 4), and distant (*n* = 7) lymph node metastases.

NA = not applicable (lesion size cannot be calculated because of diffuse type of peritoneal metastasis [irregular shape]); LN = lymph node; mets = metastases.

Data in parentheses are ranges.

### FAP and SSTR2 Expression in Tissue Microarrays of NPC

**FIGURE 6.**
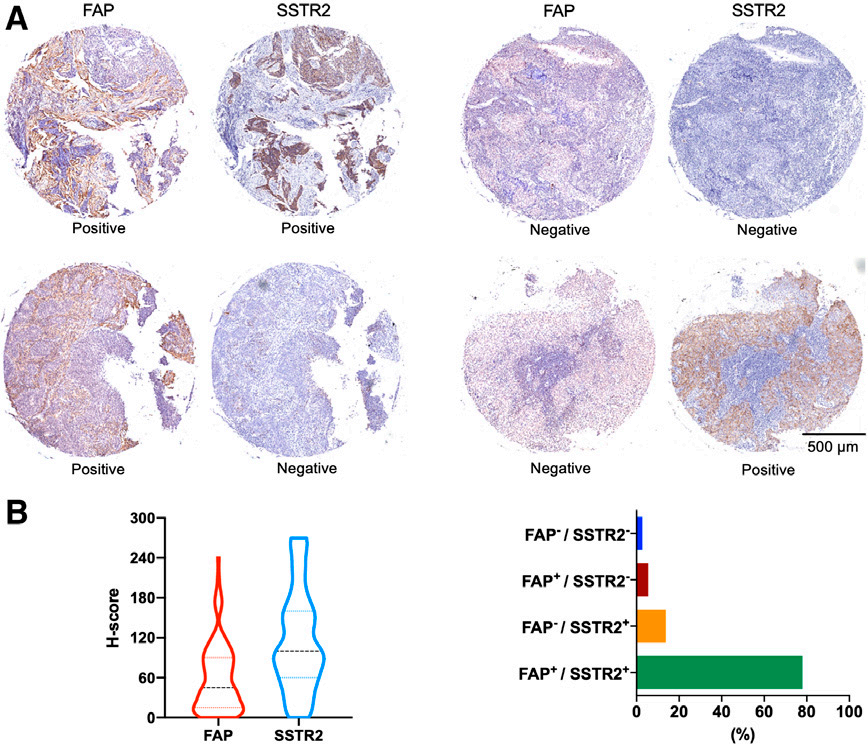
(A) Representative imaging of paired immunohistochemical staining of 110 human NPC specimens using anti-FAP and anti-SSTR2 antibodies. (B) Proportion of different H-score levels for FAP and SSTR2 expression.

We excluded 1 human NPC sample because of inadequate tumor cells. The tissue microarrays were positive for FAP or SSTR2 expression (H-score, ≥10) in most NPC samples. Remarkably, more than 77.98% of these NPC samples exhibited positivity for both markers. However, in 19.26% of the cases, discordant expression of FAP and SSTR2 was observed. This included 13.76% of cases that were FAP-negative but SSTR2-positive and 5.50% of cases that were FAP-positive but SSTR2-negative (Fig. 6).

## DISCUSSION

FAP is expressed in a wide range of tumor types, making it a promising target for cancer imaging and radionuclide therapy in recent years (4,5). Apart from neuroendocrine tumors, most other cancers, such as NPC, thyroid cancer, and breast cancer, express SSTR2 (9,14,22,23). Both proteins are located on the cell surface, reinforcing their potential as theranostic targets. This study focused on synthesis, preclinical evaluation, and pilot PET imaging of ^68^Ga-FAPI-LM3, a heterobivalent tracer designed to target both FAP and SSTR2. Our results demonstrated that this tracer exhibits a favorable safety profile and diagnostic utility in preclinical and clinical trials.

To be a good dual-targeting tracer, each binding motif of the heterobivalent molecule must retain its biologic activity. In the receptor-binding assay, the FAPI-LM3 yielded high half-maximal inhibitory concentrations for both proteins, indicating its ability to target both FAP and SSTR2 receptors. In the cell uptake and blocking assays, ^68^Ga-FAPI-LM3 showed strong binding to FAP and SSTR2. Additionally, the varied tumor internalization rates of the different compounds may influence their tumor uptake patterns (24), which necessitates further validation and warrants exploration in subsequent studies. In this study, unlabeled FAPI and DOTA-LM3 successfully blocked the binding of ^68^Ga-FAPI-LM3 to FAP and SSTR2, respectively. This result further supports the dual functionality of our tracer in specifically targeting both proteins.

In vivo experiments were conducted using small-animal PET to evaluate the performance of ^68^Ga-FAPI-LM3 in HT-1080-FAP–plus–HT-1080-SSTR2 double-positive tumor xenografts. Tumor uptake of ^68^Ga-FAPI-LM3 was significantly higher than that of ^68^Ga-FAPI and ^68^Ga-DOTA-LM3. This enhanced uptake could be attributed to an increased binding resulting from the dual-receptor targeting.

The preclinical findings suggest that the dual-receptor specificity of ^68^Ga-FAPI-LM3 allows for detecting tumors with either FAP or SSTR2 expression patterns, offering a potentially valuable tool in the diagnosis of NPC. In our preliminary clinical PET study with ^68^Ga-FAPI-LM3, it exhibited intense physiologic uptake in the blood, thyroid, pancreas, liver, kidney, and spleen. The existence of a soluble FAP form in plasma may explain the slow blood clearance in our preclinical and clinical data (25,26). Moreover, we speculate that the slow blood clearance may also be explained by the change in structure (from a monovalent to a divalent ligand) and the increase in molecular weight, which might affect the polarity of FAP/SSTR2-binding molecules (leading to increased lipophilicity). As for the high uptake in the thyroid and pancreas, similar findings were reported with ^68^Ga-labeled FAPI dimer (27,28). The reason for the increased physiologic uptake in these normal organs is still unclear. However, it should be noted that after covalent conjugation, the heterobivalent molecule is a new compound, which would show different in vivo pharmacokinetics from the monomers. Thus, it is not surprising that within normal organs and tissues, FAPI-LM3 showed distribution patterns different from either FAPI or LM3 alone.

Additionally, the uptake of ^68^Ga-FAPI-LM3 in most tumor lesions demonstrated an trend to increase from 1 to 3 h after injection. Therefore, delayed ^68^Ga-FAPI-LM3 PET/CT imaging could offer optimal lesion contrast. When ^68^Ga-FAPI-LM3 was compared with ^18^F-FDG, the standard-of-care PET tracer in oncology, we observed significantly higher uptake with ^68^Ga-FAPI-LM3 than with ^18^F-FDG in primary tumors, regional lymph node metastases, and distant metastases. As a result, ^68^Ga-FAPI-LM3 showed superiority over ^18^F-FDG in diagnosing NPC, especially in detecting lymph node, liver, bone, and peritoneal metastases. Interestingly, ^68^Ga-FAPI-LM3 also showed superiority over ^68^Ga-FAPI-46 in detecting liver and bone metastases in 1 patient with metastatic NPC.

FAP was expressed mainly on cancer-associated fibroblasts within the tumor stroma, whereas SSTR2 was expressed predominantly on tumor cells. It is also unlikely that there is dual binding because of the distance between the 2 targets: tumor cell and tumor microenvironment. There is no indication that the bivalent tracer binds simultaneously to both targets. The primary advantage of this heterobivalent molecule over its counterparts is the multivalency effect, resulting in improved tumor uptake and an increased number of effective receptors. For instance, recent research on bispecific antibodies such as PD-1/CTLA4 and PD-L1/CTLA-4 has demonstrated their potential in enhancing the effectiveness of immunotherapy (29,30). Our previous work demonstrated improved tumor uptake and retention in clinical PET studies using the heterobivalent FAPI-Arg-Gly-Asp (31). A similar strategy with heterobivalent FAPI–prostate-specific membrane antigen showed significant tumor uptake enhancement (32). In the present study, we focused on 2 promising oncologic receptors, FAP and SSTR2, which are highly expressed in most NPCs (9–11,33). The PET and biodistribution data obtained for ^68^Ga-FAPI-LM3 indicated that tumor uptake and retention were significantly improved when both targets were positively expressed in preclinical models. Compared with the monomer, which rapidly washes out from tumors, the bivalent monocular exhibits a lower dissociation rate.

The heterobivalent FAPI-LM3 comprises 2 motifs that target 2 distinct types of receptors and are covalently linked. Even if the primary motif of the heterobivalent agent detaches from the target, the secondary binding motif can still attach to the corresponding target within the tumor (34). From the perspective of clinical investigations, the main objective of the divalent molecule FAPI-LM3 was to provide a better detection rate for primary lesions and metastases of NPC instead of knowledge of FAP and SSTR2 receptor level. From this point of view, the study was successful because FAPI-LM3 PET is better than FAPI-46 PET in lesion detection. Therefore, well-designed prospective trials are needed to further investigate the diagnostic accuracy of ^68^Ga-FAPI-LM3 in primary and metastatic NPC. Additionally, the improved tumor uptake and prolonged tumor retention of FAPI-LM3 make it a suitable candidate for theranostic applications after labeling with β- or α-emitting radioisotopes for endoradiotherapy.

The dynamic nature of the tumor microenvironment and interlesion heterogeneity can, however, result in low or no expression for a specific receptor. As shown in this study using tissue microarray samples, discordant expression of FAP and SSTR2 was observed in 19.26% of NPC samples, with 13.76% of the samples being FAP-negative but SSTR2-positive and 5.50% being FAP-positive but SSTR2-negative. Under these circumstances, the heterobivalent FAPI-LM3 is superior to its monomeric counterpart for imaging of NPC. However, this superiority requires further validation through clinical data in a direct comparison of ^68^Ga-FAPI-LM3 with ^68^Ga-FAPI/^68^Ga-DOTA-LM3 PET/CT.

This study had some limitations. First, the preclinical tumor model involved transfected cell lines with extremely high FAP/SSTR2 expression. Second, the clinical study included a limited number of patients, which restricted the statistical power needed to calculate the diagnostic accuracy of ^68^Ga-FAPI-LM3 precisely. Additionally, our study lacked direct comparisons between ^68^Ga-FAPI-LM3, ^68^Ga-FAPI, and ^68^Ga-DOTA-LM3. Further investigations involving larger patient cohorts are required.

## CONCLUSION

This study demonstrated the feasibility and efficacy of ^68^Ga-FAPI-LM3, a divalent molecule for PET imaging of FAP and SSTR2, emphasizing its clinical utility for tumor detection and staging in patients with NPC. The findings contribute to understanding of dual-receptor targeting and suggest future directions for more extensive clinical studies and comparisons with other tracers.

## DISCLOSURE

This work was funded by the National Natural Science Foundation of China (82071961, 82272037), Fujian Research and Training Grants for Young and Middle-Aged Leaders in Healthcare, the Key Scientific Research Program for Young Scholars in Fujian (2021ZQNZD016), the Fujian Natural Science Foundation for Distinguished Young Scholars (2022D005), the Natural Science Foundation of Fujian Province (grant 2020J011220), Key Medical and Health Projects in Xiamen (grant 3502Z20209002), the Xiamen Key Laboratory of Radiation Oncology, the Xiamen Clinical Research Center for Head and Neck Cancer, and the 2021 National Clinical Key Specialty Grant (oncology, grant 3210013). Liang Zhao was partially funded by the China Scholarship Council (CSC). No other potential conflict of interest relevant to this article was reported.

## Notes

KEY POINTS **QUESTION:** Does a heterobivalent molecule that recognizes both FAP and SSTR2 demonstrate improved efficacy in tumor-targeting and in vivo pharmacokinetics compared with those of its corresponding monomers? **PERTINENT FINDINGS:** ^68^Ga-FAPI-LM3 showed FAP- and LM3-binding affinity both in vitro and in vivo. This study further revealed significantly higher tumor uptake of ^68^Ga-FAPI-LM3 than of ^18^F-FDG in patients with NPC. **IMPLICATIONS FOR PATIENT CARE:** The dual-receptor–targeting property of ^68^Ga-FAPI-LM3 results in improved tumor uptake and retention compared with those of its monomeric counterparts, enabling PET imaging of tumors expressing either one or both receptor types.
